# In silico intravascular optical coherence tomography (IVOCT) for quality assured imaging with reduced intervention

**DOI:** 10.1038/s41598-024-61177-1

**Published:** 2024-06-05

**Authors:** Haoyu Zhuo, Xianchen Huang, Jie Xiao

**Affiliations:** 1 School of Chemical and Environmental Engineering, College of Chemistry, Chemical Engineering and Materials Science, Soochow University, Suzhou, 215123 People’s Republic of China; 2 The Fourth Affiliated Hospital of Soochow University, Suzhou, 215123 People’s Republic of China

**Keywords:** Biomedical engineering, Chemical engineering

## Abstract

In the clinical application of intravascular optical coherence tomography (IVOCT), it is necessary to flush opaque blood during image acquisition. However, there are no specific standards for how to perform low-dose but effective flushing. In this study, computational fluid dynamics (CFD) and optical models were integrated to numerically simulate the complete process of IVOCT, which includes blood flushing with normal saline followed by image acquisition. Moreover, an intermittent injection scheme was proposed, and its advantages over the conventionally adopted scheme of continuous injection were verified. The results show that intermittent injection can significantly reduce the dosage of normal saline (reduced by 44.4%) with only a slight sacrifice of image quality (reduced by 8.7%, but still acceptable). The developed model and key findings in this work can help surgeons practice optimized IVOCT operations and potentially lead to improved designs of the IVOCT equipment.

## Introduction

**Figure 1:**
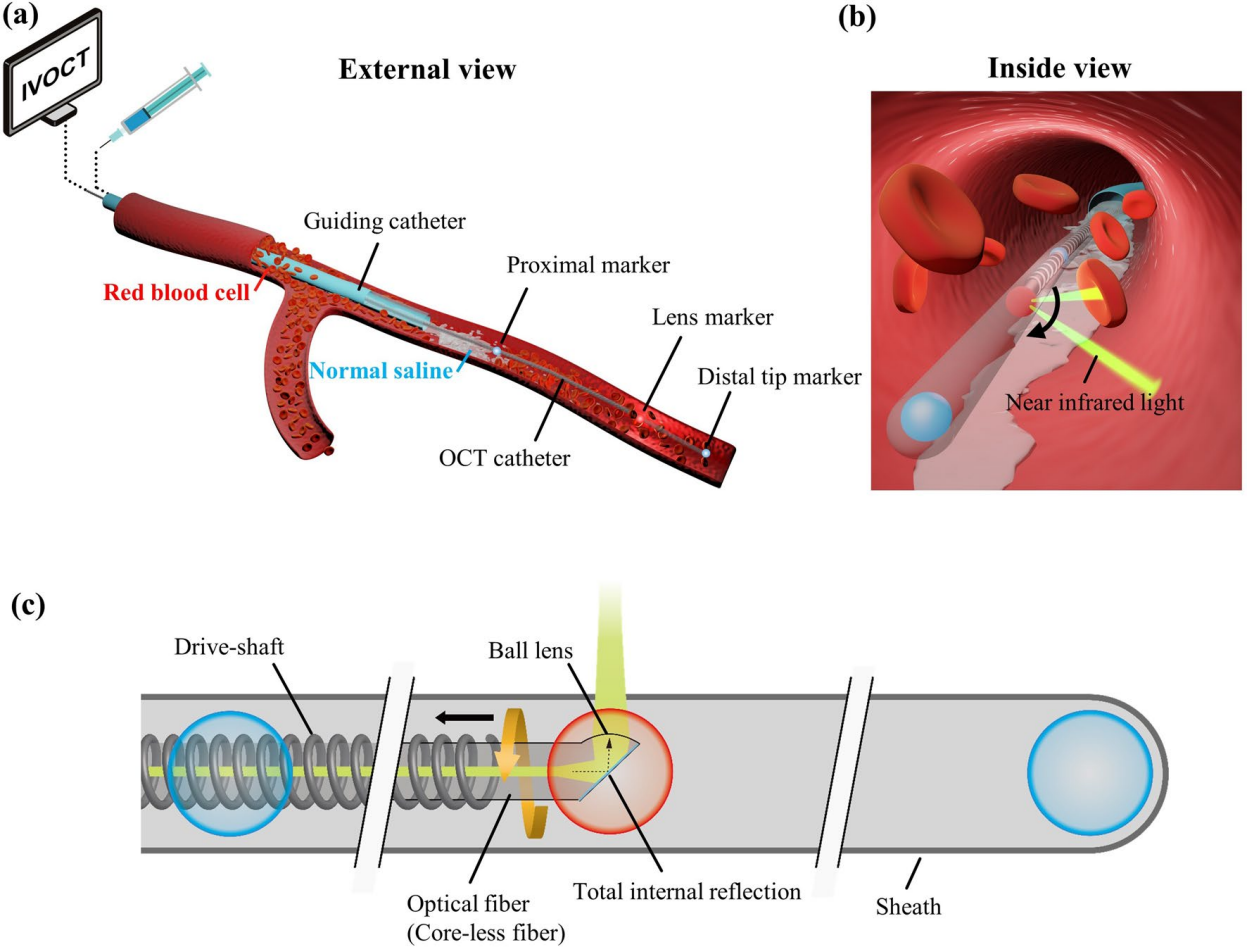
Schematic diagrams of the OCT equipment and its operation. (**a**) The overall external view of the clinical application process of OCT. (**b**) The internal view of a blood vessel showing erythrocytes that can “block” the NIR laser beam emitted from the lens. (**c**) The OCT catheter and its internal structure (modified from Fig. 2.6 in^5^).

Intravascular optical coherence tomography (OCT), also called IVOCT^1^, is an emerging and rapidly developing technology in the field of angiography. In clinical practice, doctors insert an OCT catheter into the blood vessel via interventional means. The OCT catheter is usually equipped with three opaque X-ray markers for intravascular localization (Fig. 1a). Near-infrared (NIR) light is transmitted from a single rotating optical fibre and shoots at the blood vessel wall through the imaging lens (Fig. 1b, c). The lens and internal optical fibre can be connected to the external motor through a drive-shaft for spiral retraction, and cross-sectional images of the blood vessel can be generated by measuring the echo time delay and intensity of light that is reflected or back-scattered from the internal structures of the investigated tissue during the spiral pull-back process^2^. Note that the catheter does not move after the commencement of image acquisition. It is the lens inside the OCT catheter that undergoes a spiral pullback motion. This rotation takes place inside the catheter’s transparent sheath (Fig. 1c), which effectively isolates the lens from the external blood flow environment. OCT is capable of characterizing the internal structure of the blood vessel wall with fine details due to its very high resolution, which makes this technology a powerful tool in guiding clinical research and treatment of vascular diseases.

NIR light scatters in the blood due to the mismatched refractive index of erythrocytes and plasma^3^; hence, OCT generally requires intravascular injection of contrast agent or other media through a guiding catheter to displace blood from the vascular lumen during data acquisition (Fig. 1a). If the blood on the NIR light path is not adequately cleared, amorphous bright features will appear in the OCT images. These amorphous bright features may be mistakenly diagnosed as red thrombus or blood vessel wall^4^.

Intravascular injection of contrast media for image acquisition is not used only for OCT. The discovery of X-rays by Roentgen in 1895 led to significant advancements in medical imaging technology. In 1927, Moniz obtained carotid artery X-ray photography through intravascular injection of iodine contrast. Subsequently, intravascular injection of contrast media has received extensive attention. For better X-ray imaging, the contrast agent injected into the blood vessel needs to be evenly mixed with the blood. Scholars have established many in vitro models and conducted animal experiments to investigate the mixing of contrast agent and blood in vivo to improve X-ray photography quality^6–8^. Numerical simulations have also been adopted here^9^.

Compared with X-ray photography, OCT technology is relatively new. In the early stage of its development, due to the slow image acquisition speed of the equipment, an inflated balloon had to be deployed proximal to the imaging site, blocking the upstream blood flow. Normal saline flushing could then be used to obtain clear images of a section of the downstream vessel^10^. Later, with the improvement of image acquisition speed, nonocclusive flushing became feasible. By resorting to this new technique, potential risks of vessel injury by balloon occlusion can be avoided^11^. To obtain clear images, high viscosity flushing media such as contrast agent and low molecular weight dextran have also been used to displace the erythrocytes in the blood vessel. However, unlike X-ray photography, which requires homogeneous mixing of a contrast agent and blood to achieve a satisfactory X-ray blocking effect, OCT requires that there is no residual erythrocyte that can “block” the path of NIR light in the lumen. Therefore, previous research methods and results cannot be directly adopted to the study of OCT.

The selection of flushing medium (e.g., a contrast agent, low molecular weight dextran, or normal saline) is a crucial decision-making step in the OCT medical procedure. This choice must be made with two key factors simultaneously taken into account: the blood flushing efficiency and the potential risk. It is widely accepted that a contrast agent can outperform normal saline in terms of flushing efficiency due to its higher viscosity^12–14^. However, it’s also important to note that the use of high-viscosity contrast agents poses a risk of contrast-induced nephropathy (CIN), which must be avoided^15–17^. In fact, there is no universal solution for flushing medium selection, as the optimal choice depends on the specific clinical conditions of the individual patient^17^. For patients with healthy kidneys, contrast agents may be preferred due to their superior flushing rates, leading to better image quality. However, in patients with renal impairment, the use of normal saline or low molecular weight dextran may be more appropriate so that the risk of CIN can be minimized. Also note that, compared with normal saline, low molecular weight dextran may pose a risk of allergic reactions^17^. Normal saline has long been recognized as a physiologically compatible flushing medium that can not only maintain body fluid balance, but also minimize the risk of adverse reactions^16^. Due to its reliability and versatility, normal saline has been widely used in clinical practices^13–17^.

To minimize adverse effects, it is necessary to reduce the dosage of flushing media as much as possible, especially for the contrast agent that can cause CIN. Some scholars have proposed a simple formula to calculate the needed volume of contrast agent to reduce the dosage used for each image acquisition while maintaining satisfactory image quality^18^. Alternatively, commercial systems have been used to control the injection dosage of contrast agent to achieve a similar goal^19^. For normal saline, a relatively mild solution, excessive injection can lead to vascular damage as well, especially for patients with peripheral artery disease (PAD). Sometimes multiple OCT scans have to be conducted to explore multiple sections of blood vessels in the same patient, which will lead to excessive flushing media entering the circulatory system through the guiding catheter. Under such conditions, much attention should be given to flushing media injection to avoid overdose.

Clinical application of normal saline as flushing media has received less attention compared to the contrast agent. In practice, its injection dosage is mostly based on experience or is simply equivalent to the amount of contrast agent used, which is obviously not a scientific approach. To pursue appropriate injection conditions in the clinical application of OCT using normal saline as flushing media, further theoretical and clinical investigations are needed. The characteristics of the OCT signal for the mixture of blood and normal saline have been studied through in vitro experiments^20^. Theoretical models that correlate optical measurement and haematocrit have also been developed^21^. Current fundamental understandings, however, are not comprehensive enough to guide normal saline injection in the clinical operation of OCT.

With the support of high-performance computing (HPC), multiphysics simulation is playing an increasingly important role in a wide range of fields. In silico experiments can offer doctors invaluable data that can hardly be obtained through in vivo operations. Researchers have carried out relevant investigations on intravascular injection relying on numerical simulation, such as contrast agent injection^9,22^, normal saline injection^23–25^ and intravascular administration^26^. However, the existing numerical efforts focus only on modelling fluid flow and mixing, which is just one part of the OCT operation. The more critical image acquisition process is lacking for a comprehensive characterization of the OCT operation.

By integrating computational fluid dynamics (CFD) and newly developed optical models, this work will allow in silico OCT operations to visually track the injection of normal saline and the image acquisition process. Quantitative analyses can further be carried out to pursue improved operations that can reduce the dosage of normal saline while maintaining image quality.

## Methods

### Geometry construction

**Figure 2:**
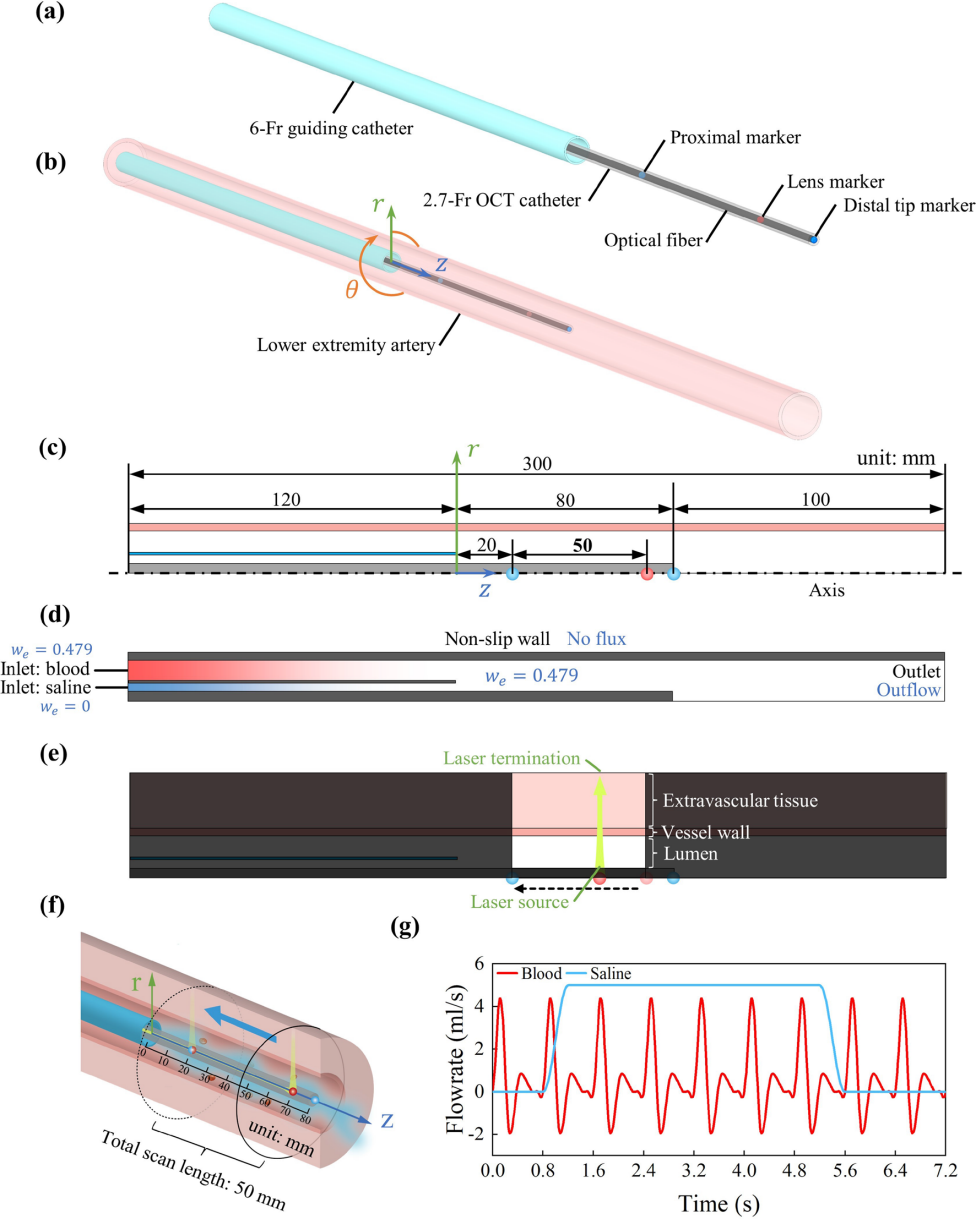
Schematic diagrams of the model geometry together with initial and boundary conditions. (**a**–**c**) Three-dimensional structure and the dimensions of the guiding catheter ($d_{o}$: 2.0 mm; $d_{i}$: 1.8 mm), OCT catheter ($d_{o}$: 0.9 mm) and blood vessel ($d_{o}$: 4.74 mm; $d_{i}$: 4.0 mm). (**d**) Boundary conditions and initial conditions of the fluid model: the black and blue fonts refer to momentum transfer and mass transfer, respectively. (**e**) Boundary conditions of the optical model. (**f**) Lens pull-back for image acquisition. (**g**) The inlet flowrates of blood and normal saline in the base case.

Schematic diagrams of the OCT model are shown in Fig. 2a–c. In this work, we focused on the study of lower extremity arteries. The lower extremity artery was simplified as a straight cylinder (see the semitransparent red tube). An OCT catheter (i.e., the transparent gray tube with a black tube inside) and a guiding catheter (blue tube) are inserted into the artery. Assuming that all tubes here share the same axial line and that the circumferential blood flow can be neglected, a two-dimensional axisymmetric model was constructed by using the COMSOL Multiphysics software^27^. As described above, OCT simulation involves two processes: blood flushing and optical image acquisition. The computational domain of the blood flushing process is shown in Fig. 2d, where the parts in black (i.e., blood vessel wall, OCT catheter and guiding catheter) are not modelled since fluids do not flow into those parts. The computational domain for optical image acquisition only includes the region where the moving lens can cover (see the unshaded region in Fig. 2e). An additional extramural tissue layer was included around the blood vessel with a thickness of 2.64 mm (resulting in a total radial length of 5 mm). The outer wall of the OCT catheter is treated as the source of the collimated NIR laser beam, which is emitted perpendicularly to the wall, passing sequentially through the vascular lumen, vessel wall and extracellular tissue. Figure 2f shows the image acquisition process, where the laser is synchronized with lens retraction. Additionally, continuous injection of flushing medium is implemented throughout the image acquisition process to flush the blood. The detailed pull-back process of the lens will be described in section “Image quality”. The current work aims to promote the use of possibly the safest flushing medium. Therefore, we specifically selected normal saline as the flushing medium in this work. It is also important to point out that our modeling methodology is general and can be used for different flusing liquids.

### Modelling for blood flushing

Blood consists of approximately 45% formed elements and 55% blood plasma by volume. The majority of the formed elements, over 99%, are erythrocytes (i.e., red blood cells, RBCs). Meanwhile, blood plasma is made up of approximately 91.5% water and 8.5% solutes by weight, with most of the solutes being proteins^28^. The simulated blood is assumed to contain 45% erythrocytes and 55% blood plasma by volume.

After the injection, mixing of blood and normal saline takes place in the blood vessel. The fluid can be treated as an incompressible fluid. Based on the blood flow conditions depicted in Fig. 2g, the maximum Reynolds number of the blood flow within a blood vessel having a diameter of 4 mm is estimated to be 770. However, during the OCT process, the injection of additional normal saline results in a significant increase in the localized Reynolds number within the vessel, reaching a maximum of 3092 at the injection flowrate depicted in Fig. 2g. It indicates a turbulent flow inside the blood vessel. In addition, the high-speed outflow of normal saline from the outlet of the guiding catheter generates a forceful jet. This jet creates a prominent separation zone inside the blood vessel with a sharp velocity gradient at the interface between the blood and jet flows^22^. To address the turbulence, the $k-\omega$ model with low-Reynolds-number wall treatment was employed. This model accurately accounts for the shearing effect between the high-velocity jet flow from the cannula and the surrounding low-velocity blood flow^22,27^.

In turbulence, the actual velocity can be regarded as the sum of the mean value $\overline{\user2{u}}$ and the fluctuation $\user2{u^{\prime}}$. The Reynolds averaged Navier–Stokes equations (RANS) were adopted to describe the fluid flow:

1 $$\frac{\partial }{\partial t}\rho + \nabla \cdot \rho \overline{\user2{u}} = 0,$$

2 $$\frac{\partial }{\partial t}\rho \overline{\user2{u}} = - \nabla \overline{p} - \left[ {\nabla \cdot \rho \user2{\overline{u}\overline{u}}} \right]p - \left[ {\nabla \cdot \left( {\overline{\user2{\tau }}^{\left( u \right)} + \overline{\user2{\tau }}^{\left( t \right)} } \right)} \right],$$

where $\rho$ is the fluid density (kg/m^3^); $\overline{\user2{u}}$ is the time-smoothed velocity (m/s); $\overline{p}$ is the time-smoothed pressure (Pa); $\overline{\user2{\tau }}^{\left( u \right)}$ is the time-smoothed viscous momentum flux (N/m^2^); and $\overline{\user2{\tau }}^{\left( t \right)}$ is the turbulent momentum flux, which is usually referred to as the Reynolds stress (N/m^2^)^29^. The viscosity of blood depends on the haematocrit (i.e., erythrocyte volume fraction in the blood). One widely adopted relation between haematocrit and viscosity was developed by Einstein^30^:

3 $$\mu = \mu_{p} \left( {1 + 2.5X_{e} } \right),$$

where $\mu$ is the viscosity of the blood and normal saline mixture (Pa s); $\mu_{p}$ is the viscosity of the plasma (Pa s), which is regarded as equivalent to the viscosity of normal saline; and $X_{e}$ is the haematocrit.

The species transport is governed by:

4 $$\frac{\partial }{\partial t}\rho w_{e} + \left( {\overline{\user2{u}} \cdot \nabla } \right)\rho w_{e} = - \nabla \cdot {\varvec{j}}_{{\varvec{e}}} ,$$

5 $${\varvec{j}}_{{\varvec{e}}} = - \rho D_{e,m} \nabla w_{e} ,$$

where $w_{e}$ is the mass fraction of erythrocytes in the mixture; ${\varvec{j}}_{{\varvec{e}}} \user2{ }$ is the diffusive flux of erythrocytes (kg/(m^2^·s)); and $D_{e,m}$ is the mass diffusion coefficient for erythrocytes in the mixture (m^2^/s). The fluid density is location- and time-dependent and can be calculated based on the volume weighted mixing law:

6 $$\rho = X_{e} \rho_{e} + \left( {1 - X_{e} } \right)\rho_{s} ,$$

where $\rho_{e}$ is the erythrocyte density (kg/m^3^) and $\rho_{s}$ is the normal saline density (kg/m^3^). The density and haematocrit of the whole blood were set to 1056 kg/m^3^ and 45%, respectively^26^. The density of normal saline is set to 1000 kg/m^3^. By applying this formula, the density of erythrocytes can be calculated as 1124.4 kg/m^3^.

The relationship between erythrocyte mass fraction $w_{e}$ and volume fraction $X_{e}$ is:

7 $$w_{e} = \frac{{X_{e} \rho_{e} }}{\rho }.$$

According to this equation, the mass fraction of erythrocytes in whole blood can be calculated as 0.479. We estimated the diffusion coefficient of erythrocytes in normal saline according to the Stocks-Einstein equation:

8 $$D_{e,m} = \frac{kT}{{3\pi d_{e} \mu }},$$

where $k$ is the Boltzmann constant; $T$ is the absolute temperature (K); and $d_{e}$ is the particle diameter (m). A red blood cell is a biconcave-shaped disk and has a volume of approximately 90 µm^3^. By using its equivalent volume diameter as the particle diameter in the above equation, the diffusion coefficients of erythrocytes in whole blood and normal saline are 4.27 × 10^–14^ m^2^/s and 9.08 × 10^–14^ m^2^/s, respectively. The Peclet Number, defined as the ratio of the convective to diffusive rate of mass transport, was calculated to be approximately 10^11^ during this process. This large Peclet number indicates that the dominant mass transport mechanism in this process is advection.

The boundary and initial conditions adopted in the blood flushing simulation are shown in Fig. 2d. Nonslip and no-flux boundary conditions were applied on the blood vessel wall as well as the inner and outer walls of the guiding and OCT catheters. Note that vessel deformation was neglected in the current study. In future, it can be modelled by the fluid–structure interaction (FSI) approach, where the biomechanical characteristics of the elastic vessel wall should be specified. The inlet flow rate of blood was determined using literature data^31^. The flow rate curves for blood and normal saline are given in Fig. 2f. Each blood flow cycle was defined as 0.8 s, and a total of 9 cycles (7.2 s in total) were simulated. In clinical practice, injections are typically administered either by manually pushing the syringe at a constant rate or by employing an automatic syringe to ensure a constant injection rate. In our simulation, the injection of normal saline commences from the second cycle and is subsequently sustained at a consistent rate for a specific duration.

The blood vessel was initially filled with whole blood, with an RBC mass fraction of 0.479, corresponding to a whole blood haematocrit of 0.45. The erythrocyte mass fraction for the blood inlet flow was also 0.479. At the inlet of the normal saline, the erythrocyte mass fraction was set to 0. At the outlet, a pressure boundary condition of 0 Pa was imposed for the flow field, while an outflow boundary condition was applied for mass transport.

### Modelling for optical image acquisition

The lens within the OCT catheter reflects the collimated NIR laser beam transmitted by the instrument through optical fibre. This laser beam will be continuously scattered and absorbed along the path by human tissues, causing its irradiance to attenuate, which can be described by Lambert‒Beer’s law^32^:

9 $$E\left( r \right) = E_{0} e^{{ - \varphi_{t} r}} ,$$

where $E\left( r \right)$ is the irradiance of the collimated NIR laser beam after travelling through the media over a distance $r$ (W/m^2^); $E_{0}$ is the irradiance of the incident collimated NIR laser beam (W/m^2^); and $\varphi_{t}$ is the total attenuation coefficient (1/mm). In the case of nonuniform tissue, the attenuation coefficient varies with position. The aforementioned equation becomes:

10 $$E\left( r \right) = E_{0} e^{{ - \mathop \smallint \limits_{0}^{r} \varphi_{t} \left( x \right)dx}} .$$

During the scattering of the collimated NIR laser beam, a portion of the scattered light is reflected back to the lens and transmitted back through the optical fibre, ultimately being detected by the instrument as an electronic signal. The intensity of this signal is directly influenced by the local refractive index and the backscattering characteristics of the tissue^33^. The signal intensity at different locations can be described according to the following equation:

11 $$I\left( r \right) = \beta \alpha \varphi_{t} \left( r \right)E_{0} e^{{ - 2\mathop \smallint \limits_{0}^{r} \varphi_{t} \left( x \right)dx}} ,$$

where $I\left( r \right)$ is the backscattered irradiance density (i.e., irradiance per unit depth) of collimated NIR laser beam at position $r$ (W/(m^2^·mm)), equivalent to the resulting OCT signal intensity; $\alpha$ is a fixed fraction representing the proportion of attenuated light backscattered; the product of $\alpha$ and $\varphi_{t}$ is also called the back scattering coefficient $\varphi_{b}$; and $\beta$ is a conversion factor representing the digitization efficiency of the detector. Both $\alpha$ and $\beta$ were set to 1 in this study. This model guarantees that the sum of the energy of all scattered light is equivalent to the initial energy of the laser, in compliance with the law of conservation of energy.

The optical properties of different human tissues exhibit variations, requiring the determination of specific optical coefficients for each region during optical simulation. The light travels in three types of media: the mixture of blood and normal saline within the vascular lumen, the vessel wall, and the extracellular tissue.

The attenuation coefficient for the light travelling in the fluid is:

12 $$\varphi_{t} = \varphi_{a} + \varphi_{s} \left( {1 - \delta } \right),$$

where $\varphi_{a}$ is the absorption coefficient (1/mm); $\varphi_{s}$ is the scattering coefficient (1/mm); and $\delta$ is the scattering anisotropy coefficient. All coefficients are dependent on the haematocrit and the specific wavelength of the NIR light employed in the analysis. These coefficients can be quantified as^21^:

13 $$\varphi_{a} = \frac{{X_{e} }}{{45{\text{\% }}}}\varphi_{{a,{ }X_{e} = 45{\text{\% }}}} ,$$

14 $$\varphi_{s} = \frac{{\left( {1 - X_{e} } \right)}}{{\left( {1 - 45{\text{\% }}} \right)}}\frac{{X_{e} }}{{45{\text{\% }}}}\varphi_{{s,X_{e} = 45{\text{\% }}}} ,$$

where $\varphi_{{a, X_{e} = 45\% }}$ and $\varphi_{{s,X_{e} = 45\% }}$ are the absorption coefficient and scattering coefficient of the whole blood, respectively, whose haematocrit is 45%. After travelling through the vessel lumen, the laser beam penetrates into the blood vessel wall and then extramural tissues. The blood vessel wall can be divided into three layers: intima, media, and adventitia, whose optical coefficients are listed in Table 1.

**Table 1:** Optical coefficients of different media for the 1300 nm laser beam.

Optical coefficient	Whole blood ($X_{e} = 45\%$)	Blood vessel wall			Extramural tissue
		Intima	media	Adventitia	
$\varphi_{a}$	0.19	–	–	–	–
$\varphi_{s}$	45.36	–	–	–	–
$\delta$	0.9779	–	–	–	–
$\varphi_{t}$	1.1925	0.67	0.48	0.53	0.5
$\alpha$	1	–	–	–	–
$\beta$	1	1	1	1	–
$\varphi_{b}$	1.1925	0.73	0.69	0.68	0.5
References	^21^	^34,34^			/

To visualize the signal intensity, the intensity values calculated by Eq. (11) were further converted to grayscale values:

15 $$G\left( r \right) = 255\frac{I\left( r \right)}{{I_{max} }},$$

where $G\left( r \right)$ is the grayscale value between 0 and 255 and $I_{max}$ is the maximum achievable OCT signal intensity. It was taken as the signal at the proximal location to the light source (i.e., OCT catheter wall) under a whole blood environment, which gives:

16 $$I_{max} = { }\alpha \beta E_{0} \varphi_{{t,X_{e} = 45{\text{\% }}}} ,$$

where $\varphi_{{t,X_{e} = 45{\text{\% }}}}$ is the total attenuation coefficient of the whole blood (1/mm). The total radial length considered in this study is 5 mm. Consequently, a one-dimensional ‘image’ with a length of 5 mm can be obtained, which is further rotated to generate a two-dimensional OCT cross-sectional image.

### Quantification of image quality and degree of intervention

#### Image quality

In clinical OCT applications, there is a time delay between the injection of normal saline and the initiation of image acquisition. This delay refers to the time needed for the normal saline to sufficiently flush the blood in the vascular lumen before the start of image acquisition. It is important to note that, conventionally, the injection of normal saline maintains a constant flow rate throughout the lens pull-back process. In the image acquisition simulation, the pull-back speed and total scanning time are set to 20 mm/s and 2.5 s, respectively, corresponding to a total pull-back and scanning length of 50 mm. The time step for cross-sectional image acquisition is set to 0.01 s, which leads to a collection of 251 cross-sectional images in total, with two consecutive cross sections 0.2 mm away from each other.

The starting time of image acquisition can be determined based on the quality of the first cross-sectional OCT image. A satisfactory OCT cross-sectional image can be called a clear image frame (CIF), which can clearly show the boundary between the lumen and the vessel wall to a certain extent^35^. However, the determination of this certain extent is based on the experience of doctors.

Since the OCT cross-sectional image needs to display discernable vessel walls’ boundary in order to clearly display the vascular structure, it is necessary to retain the light emitted from the OCT catheter as much as possible. The residual irradiance of the collimated NIR laser beam that can finally reach the inner wall of the blood vessel was recorded as $E_{w}$, which is expected to be as high as possible. In this work, we defined a residual irradiance ratio, i.e., the ratio of the residual irradiance to the initial irradiance:

17 $$M = E/E_{0} ,$$

where $E$ becomes $E_{w}$ at the inner wall of the blood vessel. $M_{w}$ is defined as the residual irradiance ratio at the inner wall of the blood vessel. MATLAB can be used to calculate $M$. It has a value between 0 and 1, where 1 refers to an ideal case in which the irradiance from the source can reach the vessel wall completely. For each cross-sectional image, one $M_{w}$ can be obtained. In this way, quantitative evaluation of the quality of each cross-sectional image becomes possible. When $M_{w}$ is higher than a specified threshold value, this image is a CIF. Note that the threshold value, which was set to 0.75 in this work, can be adjusted based on the expected image quality.

It should be pointed out that, in one pull-back process, there are many OCT cross-sectional images, whose quality contribute to the overall quality of the 3D image of the section of blood vessel under investigation. Reported methodology defined a clear imaging length (CIL)^36^, which is the cumulative length of the vessel that have been imaged well (i.e., containing CIFs). Although a higher CIL value indicates more CIFs in one scan, the distribution of CIFs along the scanned length cannot be reflected by this indicator. In this work, we defined a new indicator that takes into account both vessel wall clarity and the quality consistency of cross-sectional images:

18 $$\gamma = f_{1} \overline{{M_{w} }} + f_{2} \left( {1 - \sigma /0.25} \right),$$

where $f_{1}$ and $f_{2}$ are weights indicating the relative importance of two subindicators. To ensure that the indicator has a value between 0 and 1, the addition of two weights should be 1. In this work, specifically, $f_{1}$ and $f_{2}$ were set to 0.7 and 0.3, respectively, indicating that the vessel wall clarity received more attention. The average residual irradiance ratio at the vessel wall is:

19 $$\overline{{M_{w} }} = \mathop \smallint \limits_{0}^{L} M_{w} dx/L = \frac{1}{n}\mathop \sum \limits_{i = 1}^{n} M_{w, i} ,$$

where $L$ is the total pull-back length. $M_{w,i}$ is the residual irradiance ratio at the inner wall of the blood vessel for the *i*th image. In this work, there are a total of $n$ = 251 cross-sectional images in one complete OCT scan. The variance of all $M_{w}$ values is:

20 $$\sigma = \frac{1}{n}\mathop \sum \limits_{i = 1}^{n} \left( {M_{w,i} - \overline{{M_{w} }} } \right)^{2} .$$

To generate better OCT images, the indicator defined in Eq. (18) should have a larger value, as close to 1 as possible.

#### Degree of intervention

Although the damage caused by normal saline to the kidney is significantly less than that caused by a contrast agent or low molecular weight dextran, it is important to avoid the potential risks of a rapid and excessive injection of normal saline into the blood vessel. Inappropriate injection can lead to harm to the circulatory system, including vessel wall damage and increased urination during surgery. The dosage of normal saline injected throughout the OCT scan can be calculated to quantify the degree of intervention:

21 $$V = \mathop \smallint \limits_{0}^{{t_{f} }} Q\left( t \right)dt,$$

where $Q\left( t \right)$ is the injection flowrate of normal saline (mL/s) and $t_{f}$ is the total time of one complete scan (s).

Although hemodynamic shear stress has been found to be an important factor that can affect both endothelial function and phenotype^37^, the exact influences of stress on vessel health, especially at levels above or below physiological norms, are still under debate. The exact safety margins on stress are also unkonwn. Some scholars found that high wall shear stress is associated with signs of plaque instability that should be avoided^38^. However, certain studies indicated that slightly elevated shear stress on the endothelium might act as a shield against atherosclerosis^37^. Thus, in this work, the stress on the vessel wall was not selected as a criterion in evaluating the degree of intervetion. Instead, in the following results section, the physiological range of stress will be offered as a baseline in stress data analysis.

## Results and discussion

### Blood flushing

Effective blood flushing is essential for obtaining high-quality OCT images of the vascular wall, which can greatly facilitate the diagnosis of vascular diseases. It serves to minimize artifacts caused by erythrocytes within the lumen and enhances the residual irradiance of the collimated NIR laser beam, thereby improving the clarity of the blood vessel wall structure. The flow rate of normal saline for blood flushing in clinical practice should be related with the size of blood vessels and the blood flow conditions. The specific flow rate employed is conventionally determined based on the clinical experience of medical professionals, typically ranging between 5 and 10 mL/s^14,39^. It is known that an insufficient flow rate results in inadequate blood flushing, whereas an excessively high flow rate may adversely affect blood vessels. Nevertheless, a widely recognized optimal value is not available due to the absence of standardized protocols.

**Figure 3:**
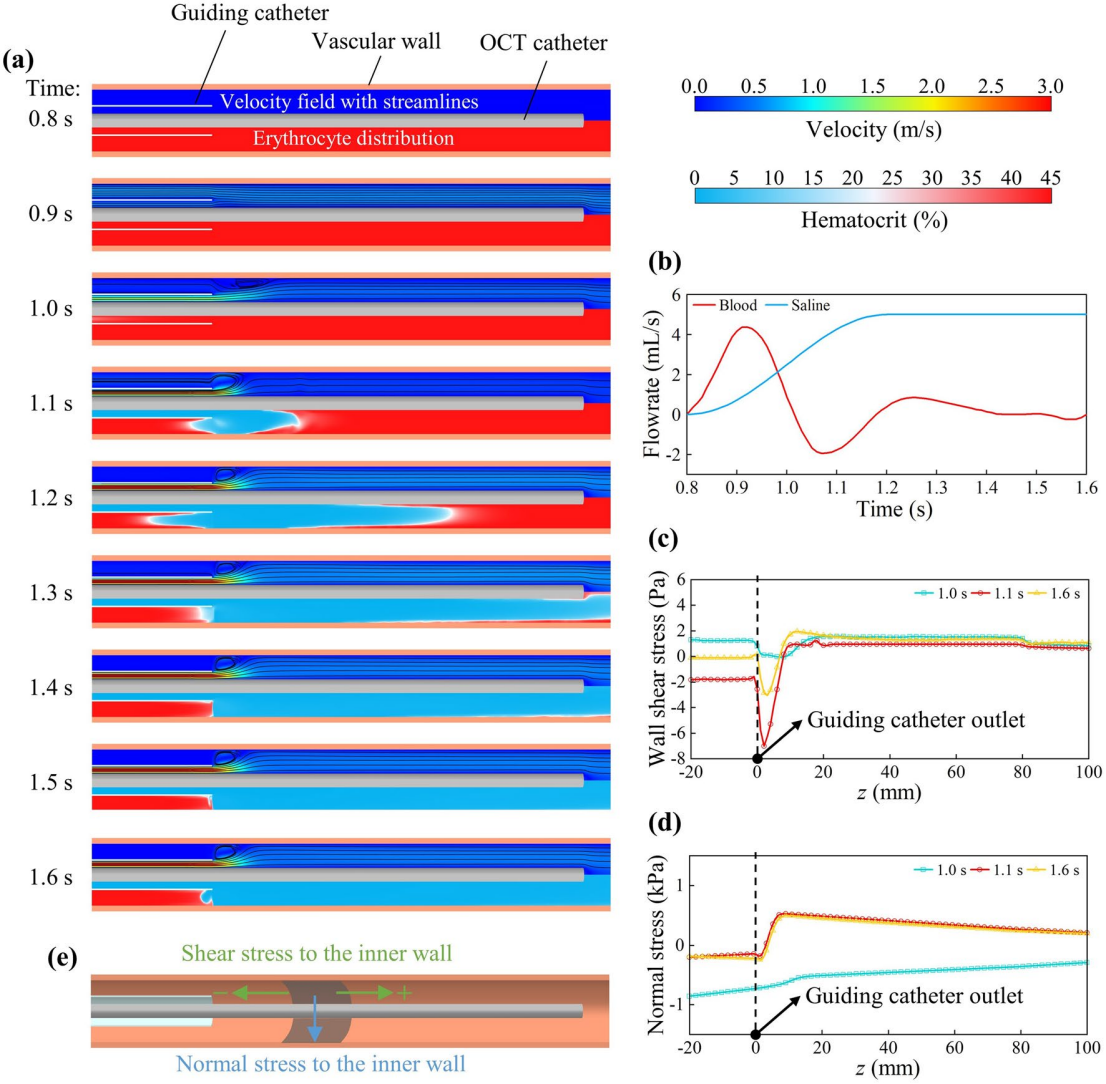
The process of flushing intravascular erythrocytes with normal saline. (**a**) Velocity field with streamlines (top half) and erythrocyte distribution (bottom half). (**b**) The flowrate of blood and normal saline in the 2nd cardiac cycle (0.8–1.6 s). (**c**) Distribution of shear stress on the inner wall of the blood vessel at 1.0 s, 1.1 s and 1.6 s. (**d**) Distribution of normal stress on the inner wall of the blood vessel at 1.0 s, 1.1 s and 1.6 s. (**e**) Schematic diagram of shear stress and normal stress on the inner vascular wall.

An in silico experiment was conducted to examine blood flushing in blood vessels with a diameter of 4 mm. The flushing process during the 2nd cardiac cycle is illustrated in Fig. 3a. Corresponding injection and blood flow conditions are given in Fig. 3b. During the systolic phase of this cardiac cycle (0.8–1.0 s), the injection of normal saline from the guiding catheter generates a forceful jet at the outlet (see the velocity field at 1.0 s in Fig. 3a, the top half plot). Note that the injection starts at 0.8 s and it takes normal saline more than 0.2 s to reach the outlet of the guiding catheter. Thus during 0.8–1.0 s, the fluid pushed out from the outlet is blood filled in the guiding catherter, and the whole blood fills completely the fluid domain throughout this period (see the erythrocyte distributions from 0.8 to 1.0 s in Fig. 3a, bottom half plots). The injection continues throughout the diastolic phase (1.0–1.6 s), accompanied by the formation of a reflux vortex close to the outlet of the guiding catheter (Fig. 3a, top half plots). As a result of the jet and reflux vortex, normal saline rapidly fills the vascular lumen after exiting the guiding catheter. The erythrocytes in the vicinity of the OCT catheter are effectively flushed at 1.3 s (Fig. 3a, the bottom half plot at 1.3 s).

The sudden injection of a large volume of fluid into the blood vessel can pose a threat to the vessel wall. The jet and reflux vortex may induce significant and sustained shear stress on the blood vessel wall. Our model is capable of predicting spatial and temporal distributions of the shear stress experienced by the vascular wall. Predicted shear stress distributions on the vascular inner wall show that the localized region close to the guiding catheter experiences intense shear stress due to the injection (Fig. 3c). In addition to the shear stress, the model can predict the evolution of normal stress exerted on the wall by the fluid (see Fig. 3d). Particularly during the blood reflux phase (e.g., 1.1 s), the combined effect of normal saline injection and blood reflux results in a peak shear stress reaching approximately 7 Pa (as indicated by the red line in Fig. 3c), which is in the negative z-direction (see the direction indicated in Fig. 3e). According to Eshtehardi et al. ^40^, a wall shear stress between 1.0 and 2.5 Pa is at the physiological range. The peak value in Fig. 3c surpasses this range and may pose a risk to vascular endothelial cells. Development of quantitative relationship between the shear and normal stresses and potential damage to the vascular wall is out of the scope of this study. The above analyses suggest, however, that it is crucial to strictly control the dosage of the injected fluid.

### Image acquisition

The quality of OCT images depends on the efficiency of blood flushing in the scanning region surrounding the lens. The presence of erythrocytes along the laser path causes a continuous decrease in irradiance, resulting in inadequate visualization of the vascular wall structure. Moreover, the scattering of laser light by erythrocytes can generate unnecessary OCT signals within the vascular lumen, which may lead to misdiagnosis.

**Figure 4:**
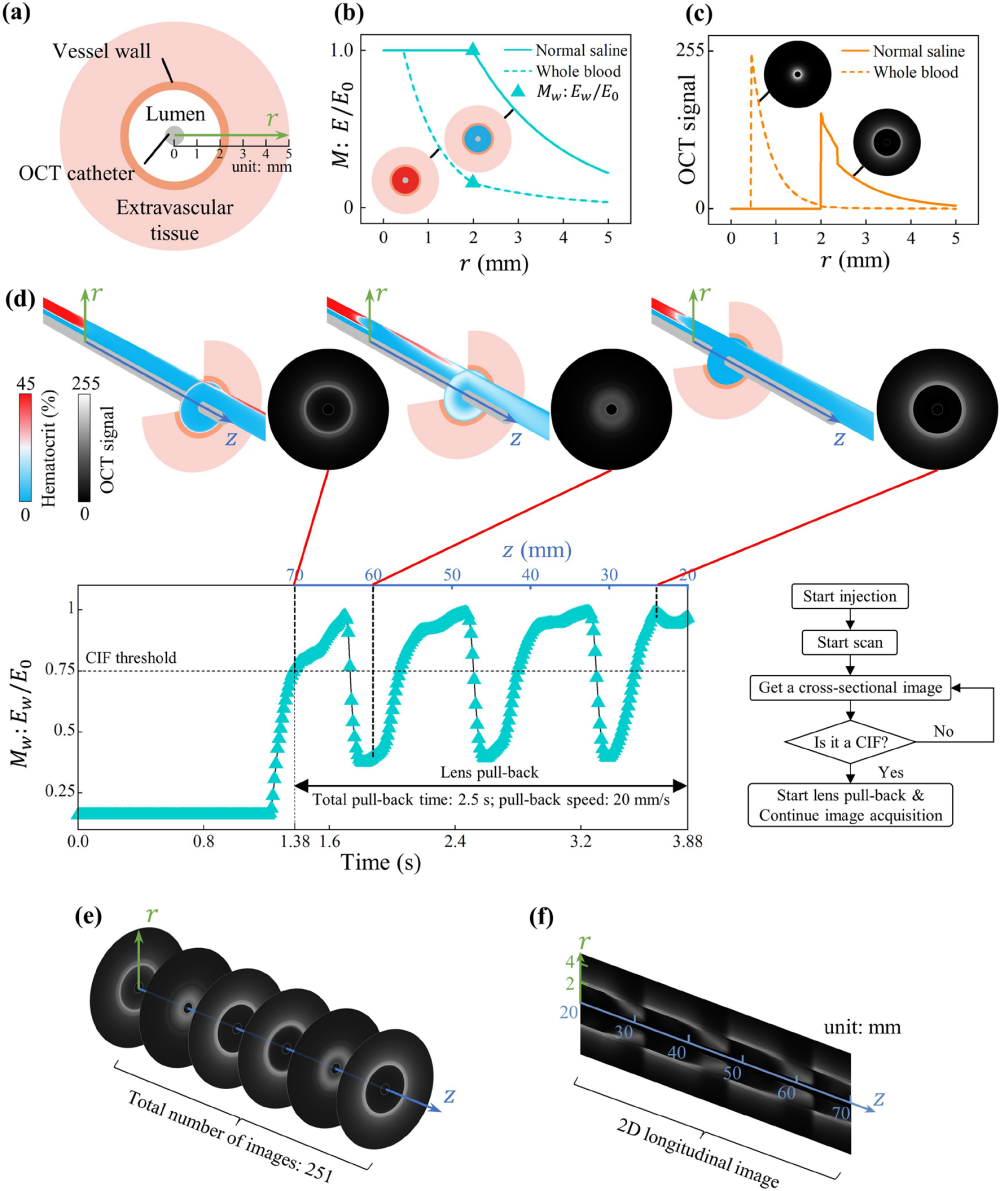
Schematic diagrams and simulation results of NIR laser beam transmission. (**a**) Cross-sectional schematic diagram of the blood vessel wall and extramural tissue. (**b**) Residual irradiance ratio (i.e., $M$) along the transmission path. (**c**) Distribution of backscattered irradiance of the NIR laser beam along the transmission path. (**d**) Residual irradiance ratio at the inner vascular wall (i.e., ${M}_{w}$) of all frames throughout the entire image acquisition process and the decision-making procedure for initiating the pull-back process. (**e**) All cross-sectional OCT images for the section of blood vessel under investigation. (**f**) Longitudinal image generated from the stack of cross-sectional images.

Two extreme scenarios are illustrated in Fig. 4a–c to delineate OCT signal visualization. Figure 4a shows a cross section of a virtual vessel to be scanned as well as its detailed dimension. Different fluids are filled in the lumen for two extreme cases. The first one involves a vascular lumen filled with normal saline, representing the optimal flushing scenario. In this case, the residual irradiance ratio within the vascular lumen (0.45 mm < $r$  ≤ 2 mm) remains constant during transmission. However, once the light reaches the blood vessel wall, this ratio starts to decrease along the path ($r$ > 2 mm, see Fig. 4b, solid line). Without RBCs in the lumen, no OCT signals can be identified in the lumen region, which implies that all irradiance can be effectively utilized to visualize the vessel wall and extramural tissues (Fig. 4c, solid line). The second scenario involves a vascular lumen filled with the whole blood, representing the worst flushing scenario. In this case, the residual irradiance ratio rapidly decreases upon entering the vascular lumen, resulting in a minimal proportion of irradiance reaching the blood vessel wall (Fig. 4b, dotted line). The majority of OCT signals are confined to the lumen region, and the small proportion of irradiance reaching the blood vessel wall results in an almost negligible OCT signal in regions of the vessel wall and extramural tissues (Fig. 4c, dotted line). As a result, in this scenario, OCT completely fails to visualize the vessel wall and extramural tissues.

During the pull-back process for image acquisition, the image quality usually lies somewhere in between the two aforementioned scenarios. To quantify the quality of all OCT cross-sectional image frames along the scanning path, we calculated the residual irradiance ratio at the inner wall of the blood vessel (i.e., ${M}_{w}$) for each image frame. They are plotted by using triangular markers in Fig. 4d. Initially, the value of this indicator is notably low due to the absence of blood flushing. However, it gradually increases due to the initiation of the normal saline injection. A decision-making algorithm was implemented to start the pull-back image acquisition process automatically when ${M}_{w}$ exceeded the CIF threshold, i.e., 0.75. The images were collected at a pull-back speed of 20 mm/s for 2.5 s, corresponding to a collection length of 50 mm.

As shown in Fig. 4d, the quality of OCT cross-sectional images exhibits periodic fluctuations throughout the acquisition process. Most frames can meet the CIF standard and the CIL in this case is 33.27 mm. According to the quality quantification method for the overall OCT image (i.e., Eq. 18) developed in this work, the overall image quality here is 0.785. There are some frames that do not meet the CIF standard, which can be attributed to the periodic blood flow bringing in new erythrocytes. Although the blood is diluted by continuously-injected normal saline, some new erythrocytes will still arrive and block the path of the laser beam, where inadequate irradiance can reach the blood vessel wall. Those regions with ${M}_{w}$ lower than 0.75 will lead to artifacts in OCT images (see for example the region close to *z* = 60 mm in Fig. 4d).

The whole section of the blood vessel can then be visualized by stacking all cross-sectional images collected sequentially along the scanning path (Fig. 4e). After 3D image reconstruction from these stacked images, we can obtain a 3D plot of the whole vessel, whose axial cut 2D plot is given in Fig. 4f. So far, we have demonstrated that our model is capable of reproducing blood flushing process and generating the quantifiable OCT image of the blood vessel under investigation. By resorting to this model, in silico experiments can then be carried out to pursue an improved OCT operation.

### In silico testing of an intermittent injection

Numerical simulation results on spatial and temporal dynamics of flow and concentration fields during the flushing process allow us to identify improvement opportunities.

**Figure 5:**
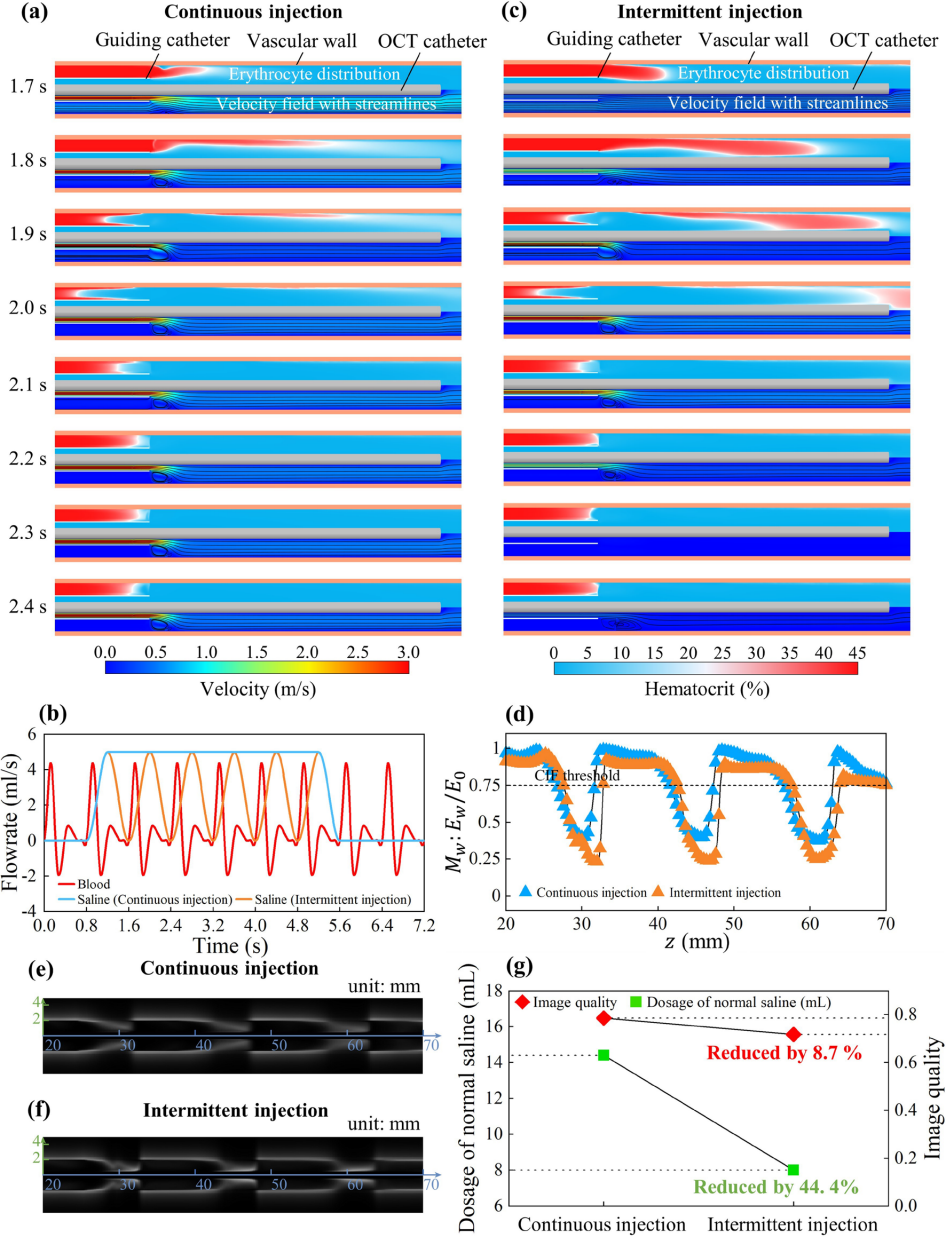
Comparison between continuous injection and intermittent injection. (**a**) Erythrocyte distribution (top half) and velocity field with streamlines (bottom half) for continuous saline injection. (**b**) Blood flowrate dynamics and waveforms of continuous and intermittent normal saline injection. (**c**) Erythrocyte distribution (top half) and velocity field with streamlines (bottom half) for intermittent injection. (**d**) Spatial distribution of residual irradiance ratio at the inner vascular wall under continuous and intermittent injections. (**e**) Longitudinal OCT image under continuous injection. (**f**) Longitudinal OCT image under intermittent injection. (**g**) Dosage of normal saline and quality of OCT images under continuous and intermittent injections.

It is known that the pulsatile nature of blood flow is attributed to the periodic contractions of the heart. One complete cardiac cycle consists of systolic and diastolic phases. The contraction of the heart during the systolic phase leads to the injection of new blood into the vessel, for which continuous normal saline injection is required to flush the incoming blood (Fig. 5a, 1.7–2.0 s). In the following diastolic phase, however, blood flow is reduced. The continuous injection of normal saline in this phase does not yield further enhancement in image quality (Fig. 5a, 2.1–2.4 s), which implies an over-flushing. The above analysis inspired us to design a cyclic intermittent injection scheme to reduce normal saline consumption. The normal saline flow rate was initially set to gradually increase and reach its peak within the first 0.4 s of a cardiac cycle. Subsequently, in the following 0.4 s, the flow rate was slowly decreased to 0 to avoid over-flushing. The waveform of this cyclic intermittent injection scheme is illustrated by the orange line in Fig. 5b, where the peak flowrate remains the same as that in continuous injection.

By comparing the velocity field and erythrocyte distribution under two schemes, it can be observed that although blood flushing efficiency is slightly deteriorated during the systolic phase for the switch to the intermittent injection (Fig. 5a, c, 1.7–2.0 s), the flushing efficiency is sufficiently good during the diastolic phase for both schemes (Fig. 5a, c, 2.1–2.4 s). This observation can be confirmed by the comparison on the distributions of the residual irradiance ratio at the blood vessel inner wall (${M}_{w}$) under two schemes (Fig. 5d). During the diastolic phase, two schemes offer similar ${M}_{w}$. Longitudinal images are also similar (Fig. 5e, f). Compared with the continuous injection, the CIL for the intermittent scheme was slightly reduced from 33.27 to 33.06 mm, which is still higher than 24 mm (i.e., a minimum value specified in the clinical study^36^). As shown in Fig. 5g, Although the overall image quality was reduced from 0.785 to 0.717, a significant reduction of normal saline consumption was achieved by switching from continuous to intermittent injection (from 14.4 to 8 ml, a 44.4% reduction). Intermittent injection should be a promising approach for improved OCT operation that can offer drastically reduced intervention with only slight sacrifice of image quality (but still acceptable).

## Summary

Development of in silico models that can numerically reproduce the OCT imaging process will greatly facilitate OCT equipment design and operational optimization. In this work, we developed a comprehensive model by coupling a CFD model with an optical model. For the first time, the process of flushing blood by using normal saline followed by image acquisition during an OCT pull-back operation was successfully simulated on a computer platform. The 3D longitudinal OCT image can be effectively reconstructed and quantitatively analyzed for image quality evaluation.

The developed models and analysis methods, although preliminary, offer the most important foundation and a useful prototype for building a powerful in silico OCT platform. By resorting to implementing in silico experiments and analyzing simulation results, we discovered an intermittent injection scheme as an alternative scheme to continuous injection. This new scheme can offer 44.4% reduction of normal saline dosage (i.e., significantly reduced intervention) with slightly reduced but still acceptable image quality.

We believe this work will lead to more efforts on OCT equipment innovations and operational optimizations in the future. To become a reliable and handy tool that can help surgeons to practice precision medication, the model has to be improved by addressing the following key questions. How to take into account patients’ physiological complexity and variations (e.g., intricate geometric variations and the elasticity of blood vessels, as well as the presence of diverse vascular lesions)? How to eliminate artifacts in the OCT image? How to deal with the impact of intravascular injection on blood circulation within the whole body and vice versa? Most importantly, clinical validations are needed. Nevertheless, this seminal work should be the first critical step on our long journey to pursuing a powerful in silico OCT platform. Moreover, similar ideas can be adopted to improve other medical imaging techniques, such as intravascular ultrasound (IVUS) and digital subtraction angiography (DSA).

## Data Availability

All data generated or analyzed during this study are available from the corresponding author on reasonable request.

## List of symbols

$d_{e}$: Equivalent volume diameter of erythrocytes (m)
$$d_{o}$$: Outside diameter (m)
$$d_{i}$$: Inside diameter (m)
$D_{e,m}$: Mass diffusion coefficient for erythrocytes in the mixture (m^2^/s)
$E$: Irradiance of the collimated NIR laser beam (W/m^2^)
$E_{0}$: Irradiance of the incident collimated NIR laser beam (W/m^2^)
$E_{w}$: Irradiance of the collimated NIR laser beam on the vessel wall (W/m^2^)
$$f_{1} , f_{2}$$: Proportion coefficients (-)
$$G$$: Grayscale value (-)
$I$: OCT signal intensity (W/(m^2^·mm))
$I_{max}$: Maximum achievable OCT signal intensity (W/(m^2^ mm))
${\varvec{j}}_{{\varvec{e}}}$: Diffusive flux of erythrocytes (kg/(m^2^ s))
$$k$$: Boltzmann constant (J/K)
$L$: Total length of the scanning area (m)
$M$: Residual irradiance ratio (-)
$M_{w}$: Residual irradiance ratio at the inner wall of blood vessel (-)
$\overline{{M_{w} }}$: Average residual irradiance ratio at the inner wall of blood vessel (-)
$n$: Total number of images (-)
$p$: Fluid pressure (Pa)
$$Q$$: Flowrate (mL/s)
$$r, z, \theta$$: Cylindrical coordinates (m, m, °)
$$t$$: Time (s)
$t_{f}$: Finish time of image acquisition (s)
$$T$$: Temperature (K)
$$\user2{u^{\prime}}$$: Velocity fluctuation (m/s)
$\overline{\user2{u}}$: Time-smoothed velocity (m/s)
$V$: Dosage of normal saline (mL)
$w_{e}$: Mass fraction of erythrocytes in the mixture (-)
$$X_{e}$$: Haematocrit (-)
$$\alpha$$: Fixed fraction (-)
$$\beta$$: Conversion factor (-)
$\rho$: Fluid density (kg/m^3^)
$\rho_{e}$: Erythrocytes density (kg/m^3^)
$\rho_{s}$: Normal saline density (kg/m^3^)
$\overline{\user2{\tau }}^{\left( u \right)}$: Time-smoothed viscous momentum flux (N/m^2^)
$\overline{\user2{\tau }}^{\left( t \right)}$: Turbulent momentum flux (N/m^2^)
$\mu$: Viscosity of blood and normal saline mixture (Pa s)
$\mu_{p}$: Viscosity of plasma (Pa s),
$\delta$: Scattering anisotropy coefficient (-)
$\varphi_{a}$: Absorption coefficient (1/mm)
$\varphi_{{a,{ }X_{e} = 45{\text{\% }}}}$: Absorption coefficient of whole blood (1/mm)
$\varphi_{b}$: Back scattering coefficient (1/mm)
$\varphi_{s}$: Scattering coefficient (1/mm)
$\varphi_{{s,{ }X_{e} = 45{\text{\% }}}}$: Scattering coefficient of whole blood (1/mm)
$\varphi_{t}$: Total attenuation coefficient (1/mm)
$\varphi_{{t,X_{e} = 45{\text{\% }}}}$: Total attenuation coefficient of whole blood (1/mm)
$$\sigma$$: Irradiation uniformity (-)
$$\gamma$$: Image quality (-)
